# Rates of delirium associated with calcium channel blockers compared to diuretics, renin-angiotensin system agents and beta-blockers: An electronic health records network study

**DOI:** 10.1177/0269881120936501

**Published:** 2020-07-08

**Authors:** Paul J Harrison, Sierra Luciano, Lucy Colbourne

**Affiliations:** 1 Department of Psychiatry, University of Oxford, Oxford, UK; 2 Oxford Health NHS Foundation Trust, Oxford, UK; 3 TriNetX Inc., Cambridge, USA

**Keywords:** Delirium, pharmacoepidemiology, antihypertensives, electronic health records, voltage-gated calcium channel

## Abstract

**Background:**

Antihypertensive drugs, especially calcium channel blockers, have been associated with differential rates of a number of neuropsychiatric outcomes. Delirium is commonly attributed to medication, including antihypertensive drugs, but delirium incidence has not been compared directly between antihypertensive drug classes.

**Methods:**

Using a federated electronic health records network of 25.5 million people aged 50 years or older, we measured rates of delirium over a two-year period in patients prescribed calcium channel blockers compared to the other main antihypertensive drug classes. Extensive propensity score matching was used to create cohorts matched for a range of demographic factors and delirium risk factors. Negative control outcomes were also measured.

**Results:**

Cohort sizes ranged from 54,000–577,000. Delirium was more common with calcium channel blockers than with renin-angiotensin system agents (~40% higher) but less common than with beta-blockers (~20% lower). These differences remained when patients with a range of other delirium risk factors were excluded, and they were not paralleled by the negative control outcomes. Comparisons between calcium channel blockers and diuretics produced inconclusive results.

**Conclusions:**

Calcium channel blockers are associated with higher rates of delirium than renin-angiotensin system agents, but lower rates compared to beta-blockers. The findings add to the list of factors which may be considered when choosing antihypertensive drug class.

## Introduction

Pharmacoepidemiological data show that antihypertensive drugs (AHTs) are associated with a reduced risk for several neuropsychiatric disorders, including dementia (Hussain et al., 2018; Rouch et al., 2015), Parkinson’s disease (Mullapudi et al., 2016) and stroke (Mukete et al., 2015). Some data suggest that, amongst AHT classes, calcium channel blockers (CCBs) and renin-angiotensin system (RAS) agents (angiotensin-converting enzyme inhibitors (ACEIs) and angiotensin II inhibitors (ARBs)) are more protective in this regard than are diuretics and beta-blockers (Marpillat et al., 2013; Mukete et al., 2015; Mullapudi et al., 2016; Rouch et al., 2015; Zhuang et al., 2016). Less extensive data also report associations between CCB and RAS agent use with functional psychiatric disorders such as psychosis (Hayes et al., 2019) and depression (Boal et al., 2016).

Delirium is a common clinical problem, especially in the elderly. Medication is an important cause, with risks well recognised for drugs such as digoxin, anti-arrhythmics and dopaminergic agents (Alagiakrishnan and Wiens, 2004; Inouye et al., 2014; Thorn et al., 2019). AHTs are also listed as risk factors for delirium, but the relative risks associated with the major AHT classes are not known. Here we used an electronic medical records network to assess whether AHT classes are associated with differential risks of delirium over a two-year period, taking advantage of a large sample size and unusually detailed data availability. We used extensive propensity score matching (Ali et al., 2019; Austin, 2011) and negative control outcomes (Arnold and Ercumen, 2016; Lipsitch et al., 2010) to reduce the confounding which affects pharmacoepidemiological studies (Davis et al., 2020; Freemantle et al., 2013; Kyriacou and Lewis, 2016). We chose CCBs as our reference AHT class for two main reasons. Firstly, compared to other AHTs, there are more data linking CCBs with neuropsychiatric outcomes, facilitating interpretation of results in the context of the existing literature. Second, voltage-gated calcium channels, the target of CCBs, are increasingly implicated in the aetiology of a range of psychiatric and neurological conditions (Heyes et al., 2015; Nimmrich and Eckert, 2013), and CCBs have been proposed for repurposing for these indications (Cipriani et al., 2016; Dubovsky, 2019; Harrison et al., 2020; Liss and Striessnig, 2019).

## Methods

TriNetX (www.trinetx.com) is a federated cloud-based network providing access to electronic medical records from multiple healthcare organisations (HCOs) comprising a mix of hospitals, primary care and specialty treatment providers. The HCOs span many geographic locations, age groups and income levels. Aggregated patient data and results from statistical analyses are provided through a browser-based interface. TriNetX has a waiver from the Western Institutional Review Board since only aggregated counts and statistical summaries of de-identified information are used; no protected health information is received and no primary data collection is performed as part of the retrospective analysis (Stapff, 2018). The present study used the TriNetX Analytics network, which allows access to data from ~61.4 million patients in 46 HCOs in the USA. The identity of individual HCOs and the contribution of each HCO to the datasets studied is not revealed; however, we do know the proportion of patients from each quadrant of the USA, and this did not differ for any of the analyses performed here (data not shown). HCOs refresh their data on average every 24 days, and data are retrieved in response to a search query submitted via the browser (see below). The available data include extensive information on demographics, diagnoses (using International Classification of Diseases, 10th revision [ICD-10] codes), medications, procedures and laboratory values. Most data were collected from 2007 onwards.

The network allows patient cohorts to be created based on defined inclusion and exclusion criteria related to diagnosis, medications, laboratory results, etc. Combinations of criteria can be used, with specified temporal relationships between them. Two cohorts thereby selected (e.g. people treated with CCBs vs people treated with diuretics) can then be compared for demographic, diagnostic and other characteristics. There is a built-in capability to propensity score match cohorts for any variables of interest (e.g. age, sex, blood pressure, prior diagnoses, previous treatment, etc.). The method as implemented by TriNetX uses greedy nearest neighbour matching with a caliper distance of 0.1 pooled standard deviations of the logit of the propensity score, to produce 1:1 matching. We considered any variable which had a standard difference between groups of less than 0.1 to be well matched (Haukoos and Lewis, 2015).

We excluded patients younger than 50 years old. To avoid possible confounding of delirium with dementia and related conditions, we also excluded anyone with a recorded prior diagnosis of dementia (ICD-10 codes: F01, F02, F03), Alzheimer’s disease (G30) or other neurodegenerative diseases (G31). All other patients (~25.5 million) were eligible to be included in a cohort, with separate pairwise analyses comparing a CCB-treated cohort with a cohort prescribed diuretics, or with a cohort prescribed RAS agents, or with a cohort prescribed beta-blockers.

Our outcome period was 2 years; exposure throughout this time was proxied by requiring each subject to have had at least 2 prescriptions for the AHT class, 2 years apart. Our outcome of interest was delirium. As coded in the network, this comprises ‘delirium due to a known physiological condition’ (F05), as well as R40.0 (‘somnolence’ or ‘drowsiness’) and R41.0 (‘disorientation, unspecified’ or ‘confusion’). The latter two categories likely capture episodes of hypoactive delirium which were not coded under F05. We also measured the F05 diagnostic category separately. To help assess residual confounding, we also compared rates of 12 negative control outcomes (i.e. outcomes for which there are neither mechanistic reasons to expect, nor evidence to indicate, any differences between CCBs and other AHTs; Arnold and Ercumen, 2016; Lipsitch et al., 2010). We also measured a range of other variables during the exposure period which might be associated with the occurrence of delirium, as described below.

Within this overall analytic framework, we created three types of cohort:

1. In initial unadjusted (‘unmatched’) analyses, we included all patients who met the inclusion criteria above. Reflecting hypertension guidelines and clinical practice (Musini et al., 2017; Whelton et al., 2018; Williams et al., 2018), choice of AHT class is influenced by a number of factors, including age, sex, race and diabetes mellitus; moreover, beta-blockers are more commonly used than the other AHT classes for indications other than hypertension. We anticipated that these factors would lead to unbalanced cohorts and with potential for significant confounding by indication; however, we considered the approach valuable to get an initial estimate, and it produces data comparable to studies in which matching is limited or absent.
2. For our main ‘matched’ analyses, we propensity score matched cohorts for age, sex, race, systolic blood pressure, body mass index (BMI), diabetes mellitus, thyroid disease, ischaemic heart disease and cerebrovascular disease, and for prior use of AHTs, anticoagulants, statins and platelet aggregation inhibitors. We also matched the cohorts for rates of various other diagnoses and treatments which could affect their subsequent risk of delirium: surgery, sepsis, alcohol use disorder, previous delirium and use of digitalis glycosides, anti-arrhythmics, dopaminergics (stimulants and anti-Parkinsonian medication), cholinergics (parasympathetic agents), sedatives/hypnotics, opioids, antidepressants, antipsychotics, gabapentinoids or adrenal steroids. Because the contribution of AHTs to delirium may differ with ageing, we also repeated the matched analyses restricting our cohorts to patients who were 70 years of age or older.
3. In the third approach, we created ‘refined’ cohorts in which we excluded all patients with a prior diagnosis of delirium, and all patients who, either previously or during the two-year outcome period had any of the following: a diagnosis of sepsis or alcohol use disorder, or prescription of digitalis glycosides, anti-arrhythmics, cholinergics or dopaminergics. Apart from these exclusion criteria, propensity score matching was carried out as for the ‘main’ cohorts. The combination of criteria used for the refined cohorts was intended to increase the proportion of delirium which is attributable to AHT medication and it results in the least confounded estimates. However, it results in smaller cohorts, with lower rates of delirium, and is less representative of the overall population.

Group comparisons for delirium and other outcomes were made using odds ratios (ORs) and 95% confidence intervals, calculated using the suite of analytic features within the TriNetX network. Our study followed the STROBE guidelines (von Elm et al., 2007).

## Results

### Unadjusted analyses

The details of the unmatched cohorts and the results are shown in Table 1. CCBs are seen to be associated with lower rates of delirium than diuretics or beta-blockers, but with a higher rate than RAS agents. However, as anticipated, there are also differences between these groups in terms of age, sex, race, blood pressure, BMI, previous AHT exposure, and diabetes mellitus, any of which could affect delirium risk. Moreover, the difference in delirium incidence seen when comparing CCBs with diuretics is paralleled by the negative control outcomes (for details, see Supplementary Material Table 1), suggesting a non-specific effect of general health or health-seeking behaviour. Finally, for each of the three comparisons, during the exposure period there are differences in a range of delirium risk factors (such as a diagnosis of sepsis or the rate of digoxin prescribing) which could further confound the results. Table 1 also shows that the data density – the average number of facts known about each patient – is high, and similar for CCB and other cohorts; this argues that any observed group differences (e.g. in rates of diagnosis or medication use) are not simply a reflection of differential data completeness.

**Table 1.** Unmatched cohorts: The incidence of delirium over a two-year period associated with calcium channel blockers (CCBs) compared to diuretics, renin-angiotensin system (RAS) agents and beta-blockers.

	CCBs vs diuretics		CCBs vs RAS agents		CCBs vs beta-blockers	
***Baseline characteristics***						
	*CCBs*	*Diuretics*	*CCBs*	*RAS agents*	*CCBs*	*Beta-blockers*
Cohort size	159,663	439,194	130,016	577,522	195,525	446,653
Age at index (y)	63.7 (11.0)	61.9 (11.2)	64.3 (11.6)	61.2 (10.5)	62.9 (11.0)	63.2 (11.3)
Sex (M:F)	50.5%:49.5%	40%:60%	42%:58%	50%:50%	44%:56%	50%:50%
Race (W, B/AA, O/NK)^a^	66%, 20%, 14%	76%, 15%, 9%	64%, 24%, 12%	77%, 11%, 12%	63%, 26%, 11%	78%, 9%, 13%
Systolic BP^b^	139 (21)	133 (20)	137 (21)	135 (20)	140 (20)	130 (20)
Diastolic BP^b^	79 (13)	78 (13)	78 (13)	78 (12)	81 (13)	75 (12)
BMI^b^	29 (6)	32 (8)	29 (7)	31 (7)	30 (7)	30 (7)
Diabetes mellitus	17%	19%	13%	24%	18%	18%
Previous exposure to AHTs	30% BB, 43% RAS	31% BB, 52% RAS	32% BB, 28% D	28% BB, 40% D	36% D, 51% RAS	33% D, 39% RAS
Data density (average facts per patient)^c^	7397	9985	8761	8605	9263	8372
***Outcomes***						
	*CCBs vs diuretics*	*Odds ratio (95% CI)*	*CCBs vs RAS agents*	*Odds ratio (95% CI)*	*CCB vs beta-blockers*	*Odds ratio (95% CI)*
Delirium (any)	1.22% vs 1.46%	**0.83 (0.79**–**0.88)**	1.89% vs 1.12%	**1.70 (1.62**–**1.78)**	1.02% vs 1.51%	**0.67 (0.64**–**0.71)**
Delirium (F05 only)	0.19% vs 0.26%	**0.75 (0.66**–**0.85)**	0.32% vs 0.14%	**2.19 (1.95**–**2.46)**	0.14% vs 0.28%	**0.49 (0.43**–**0.56)**
Negative control outcomes^d^		**0.84 (0.79**–**0.89)**		0.99 (0.91–1.06)		**1.17 (1.06**–**1.27)**
***Factors associated with delirium risk during exposure period***						
Alcohol use disorder	1.37% vs 1.38%	0.99 (0.94–1.04)	1.52% vs 1.18%	**1.30 (1.23**–**1.36)**	1.26% vs 1.33%	**0.94 (0.90**–**0.99)**
Sepsis	1.06% vs 1.57%	**0.67 (0.64**–**0.71)**	1.91% vs 0.92%	**2.10 (2.00**–**2.21)**	0.89% vs 1.50%	**0.59 (0.56**–**0.62)**
Surgery	53.6% vs 58.5%	**0.82 (0.81**–**0.83)**	55.6% vs 56.1%	**0.98 (0.97**–**0.99)**	57.7% vs 52.1%	**1.26 (1.24**–**1.27)**
Digitalis glycosides	0.87% vs 2.64%	**0.32 (0.31**–**0.34)**	1.94% vs 1.61%	**1.21 (1.16**–**1.26)**	0.69% vs 3.30%	**0.20 (0.19**–**0.22)**
Anti-arrhythmics	17.8% vs 20.6%	**0.83 (0.82**–**0.84)**	21.9% vs 17.7%	**1.31 (1.29**–**1.33)**	15.7% vs 22.0%	**0.66 (0.65**–**0.67)**
Dopaminergics	3.76% vs 4.60%	**0.81 (0.79**–**0.83)**	4.34% vs 3.69%	**1.18 (1.15**–**1.22)**	3.60% vs 4.52%	**0.79 (0.77**–**0.81)**
Cholinergics	8.2% vs 9.4%	**0.86 (0.84**–**0.88)**	9.97% vs 8.01%	**1.27 (1.24**–**1.30)**	6.2% vs 9.1%	**0.66 (0.64**–**0.67)**

AHT: antihypertensive drug; BMI: body mass index: BP: blood pressure; CI: confidence interval.

a W: white; B/AA: black or African American; O/NK: other or not known.

b Most recent value before exposure period.

c Comprising diagnoses, procedures, medications, laboratory results and vital signs.

d Mean of 12 negative control outcomes. See Supplementary Material Table 1 for details.

### Analyses of ‘matched’ cohorts

For the main analyses, we propensity score matched cohorts extensively for demographic and delirium risk factors, as noted above. This process was successful for all variable, with all standard differences being less than 0.1 except where noted. The details of the resulting cohorts, and the findings, are shown in Table 2. The results largely mirror those of the unmatched cohorts, with CCBs associated with lower rates of delirium than diuretics or beta-blockers, but a higher rate than RAS agents. Differences in the negative control outcomes were also comparable (see Supplementary Material Table 2), as was the differential incidence of delirium risk factors during the two-year period.

**Table 2.** Matched cohorts: Incidence of delirium over a two-year period associated with calcium channel blockers (CCBs) compared to diuretics, renin-angiotensin system (RAS) agents and beta-blockers.

	CCBs vs diuretics		CCBs vs RAS agents		CCBs vs beta-blockers	
***Baseline characteristics***						
	*CCBs*	*Diuretics*	*CCBs*	*RAS agents*	*CCBs*	*Beta-blockers*
Cohort size	157,944	157,944	127,919	127,919	167,037	167,037
Age at index (y)	63.7 (11.0)	64.0 (11.3)	64.2 (11)	64.3 (11.2)	63.1 (11.0)	63.3 (11.2)
Sex (M:F)	50%:50%	50%:50%	42%:58%	42%:58%	45%:55%	45%:55%
Race (W, B/AA, O/NK)^a^	66%, 20%, 14%	67%, 19%, 14%	65%, 23%, 12%	65%, 22%, 13%	70%, 18%, 12%	70%, 18%, 12%
Systolic BP^b^	138 (20.8)	136 (20.8)	137 (21)	137 (21)	139 (20)^c^	136 (20)^c^
Diastolic BP^b^	79 (12.8)	78 (13.0)	78 (13)	78 (13)	80 (13)^d^	78 (13)^d^
BMI^b^	29 (6.3)	29 (6.8)	29 (7)	29 (7)	30 (7)	31 (7)
Diabetes mellitus	17%	18%	13%	13%	16%	19%
Previous exposure to AHTs	30% BB, 44% RAS	30% BB, 44% RAS	32% BB, 28% D	32% BB, 28% D	35% D, 48% RAS	35% D, 47% RAS
***Outcomes***						
	*CCBs vs diuretics*	*Odds ratio (95% CI)*	*CCBs vs RAS agents*	*Odds ratio (95% CI)*	*CCBs vs beta-blockers*	*Odds ratio (95% CI)*
Delirium (any)	1.22% vs 1.50%	**0.81 (0.76**–**0.86)**	1.85% vs 1.24%	**1.51 (1.41**–**1.61)**	1.03% vs 1.31%	**0.78 (0.74**–**0.84)**
Delirium (F05)	0.19% vs 0.27%	**0.69 (0.59**–**0.79)**	0.31% vs 0.17%	**1.87 (1.58**–**2.21)**	0.14% vs 0.23%	**0.62 (0.53**–**0.73)**
Negative control outcomes^e^		**0.87 (0.83**–**0.92)**		0.99 (0.91–1.06)		1.07 (1.00–1.14)
***Factors associated with delirium risk during exposure period***						
Alcohol use disorder	1.35% vs 1.54%	**0.88 (0.83**–**0.93)**	1.51% vs 1.14%	**1.33 (1.24**–**1.43)**	1.21% vs 1.30%	**0.93 (0.87**–**0.99)**
Sepsis	1.04% vs 1.62%	**0.64 (0.60**–**0.68)**	1.87% vs 1.00%	**1.89 (1.76**–**2.02)**	0.90% vs 1.20%	**0.75 (0.70**–**0.80)**
Surgery	53.5% vs 56.9%	**0.87 (0.86**–**0.88)**	55.4% vs 56.1%	**0.97 (0.96**–**0.99)**	56.2% vs 54.0%	**1.09 (1.08**–**1.11)**
Digitalis glycosides	0.88% vs 2.06%	**0.42 (0.39**–**0.45)**	1.94% vs 1.74%	**1.12 (1.05**–**1.18)**	0.81% vs 1.62%	**0.49 (0.46**–**0.53)**
Anti-arrhythmics	17.8% vs 20.3%	**0.85 (0.83**–**0.86)**	21.9% vs 19.4%	**1.16 (1.14**–**1.18)**	16.2% vs 19.1%	**0.82 (0.80**–**0.83)**
Dopaminergics	3.78% vs 4.02%	**0.93 (0.90**–**0.97)**	4.34% vs 3.66%	**1.19 (1.15**–**1.24)**	3.85% vs 4.06%	**0.95 (0.92**–**0.98)**
Cholinergics	8.19% vs 9.09%	**0.89 (0.87**–**0.91)**	9.90% vs 8.60%	**1.17 (1.14**–**1.20)**	6.38% vs 8.52%	**0.73 (0.71**–**0.75)**

AHT: antihypertensive drug; BB: beta-blocker; BMI: body mass index: BP: blood pressure; CI: confidence interval; D: diuretic.

a W: white; B/AA: black or African American; O/NK: other or not known.

b Most recent value before exposure period.

c Standard difference=0.15.

d Standard difference=0.16.

e Mean of 12 negative control outcomes. See Supplementary Material Table 2 for details.

We repeated the matched cohort analysis in the subgroup of people aged 70 years or over. Cohort sizes were ~91,000 (CCBs versus diuretics), ~71,000 (CCBs versus RAS agents) and ~99,000 (CCBs versus beta-blockers). Results were similar to the matched analysis of the full cohort. Thus, in the over 70s, CCBs were associated with less delirium than diuretics (OR 0.84 (079–0.90)) or beta-blockers (OR 0.82 (077–0.89)), but with more delirium than RAS agents (OR 1.28 (1.18–1.38)).

### Analyses of ‘refined’ cohorts

The refined cohorts excluded anyone with a past history of delirium, or who previously or during the two-year period received certain diagnoses and treatments known to increase risk of delirium (see Methods). As anticipated, this resulted in much smaller cohorts (especially due to exclusion of patients receiving anti-arrhythmics), and lower rates of delirium compared to the unadjusted and main analyses. The results are shown in Table 3. The CCB vs diuretic comparison for delirium is no longer significant, but the group difference is still observed for negative control outcomes (Supplementary Material Table 3) and for the incidence of surgery. The higher rate of delirium with CCBs compared to RAS agents remains, and is not paralleled by negative control outcomes nor rate of surgery. Finally, CCBs continue to be associated with less delirium than beta-blockers (albeit not for the F05 category alone), even though the rate of surgery was higher in the group taking CCBs; negative control outcomes were the same between CCB and beta-blocker groups.

**Table 3.** Refined cohorts: Incidence of delirium over a two-year period associated with calcium channel blockers (CCBs) compared to diuretics, renin-angiotensin system (RAS) agents and beta-blockers.

	CCBs vs diuretics		CCBs vs RAS agents		CCBs vs beta-blockers	
***Baseline characteristics***						
	*CCBs*	*Diuretics*	*CCBs*	*RAS agents*	*CCBs*	*Beta-blockers*
Cohort size	76,083	76,083	54,224	54,224	80,463	80,463
Age at index (y)	63.6 (11.1)	63.9 (11.3)	63.9 (11.6)	64.0 (11.4)	63.0 (11.0)	63.2 (11.3)
Sex (M:F)	50%:50%	49%:51%	41%:59%	41%:59%	45%:55%	44%:56%
Race (W, B/AA, O/NK)^a^	63%, 20%, 17%	64%, 19%, 17%	61%, 25%, 14%	61%, 25%, 14%	68%, 17%, 15%	68%, 17%, 15%
Systolic BP^b^	139 (20)	138 (20)	139 (20)	138 (20)	139 (20)^c^	136 (20)^d^
Diastolic BP^b^	80 (12)	80 (12)	80 (12)	80 (12)	81 (12)	79 (12)^d^
BMI^b^	29 (6)	30 (7)	30 (7)	30 (7)	30 (7)	31 (7)
Diabetes mellitus	15%	16%	11%	10%	15%	17%
Previous exposure to AHTs	26% BB, 45% RAS	26% BB, 44% RAS	26% BB, 25% D	26% BB, 25% D	33% D, 46% RAS	33% D, 47% RAS
***Outcomes***						
	*CCBs vs diuretics*	*Odds ratio (95% CI)*	*CCB vs vs RAS agents*	*Odds ratio (95% CI)*	*CCBs vs beta-blockers*	*Odds ratio (95% CI)*
Delirium (any)	0.63% vs 0.68%	0.93 (0.82–1.06)	0.74% vs 0.53%	**1.41 (1.21**–**1.64)**	0.56% vs 0.64%	**0.87 (0.76**–**0.98)**
Delirium (F05)	0.07% vs 0.09%	0.80 (0.55–1.17)	0.11% vs 0.05%	**1.93 (1.24**–**3.01)**	0.05% vs 0.07%	0.73 (0.49–1.08)
Negative control outcomes^e^		**0.90 (0.87**–**0.94)**		1.00 (0.93–1.07)		1.06 (1.00–1.12)
***Factors associated with delirium risk during outcome period***						
Surgery	46.1% vs 47.7%	**0.94 (0.92**–**0.96)**	46.6% vs 46.9%	0.99 (0.96–1.01)	48.9% vs 43.7%	**1.24 (1.21**–**1.26)**

AHT: antihypertensive drug; BB: beta-blocker; BMI: body mass index: BP: blood pressure; CI: confidence interval; D: diuretic.

a W: white. B/AA: black or African American. O/NK: other or not known.

b Most recent value before exposure period.

c Standard difference=0.13.

d Standard difference=0.16.

e Mean of 12 negative control outcomes. See Supplementary Material Table 3 for details.

In summary, the three levels of analysis indicate that there is a robust increase in delirium incidence associated with CCBs compared to RAS agents, but a lower incidence with CCBs compared to beta-blockers. CCBs also appeared to have a lower risk than diuretics, but the results were not consistent nor readily interpretable, reflecting confounding factors between the groups which could not be removed.

## Discussion

Delirium is often attributed to medication, and AHTs are amongst the implicated drugs (Alagiakrishnan and Wiens, 2004; Inouye et al., 2014). Our data suggest that not all AHTs are the same in this regard. In cognitively healthy people over a two-year period, CCBs are associated with a higher rate of delirium than RAS agents, but a lower rate than beta-blockers. The unusually large sample size, and richness of data available, allowed us to examine and control for many possible confounders, including confounding by indication (i.e. factors which influence choice of AHT class), and for other risk factors for delirium (e.g. sepsis, surgery, digitalis glycosides). We did this using propensity score matching for a wide range of factors and, in the refined cohorts, by excluding people with diagnoses and medications which are strong risk factors for delirium. The use of negative control outcomes provided an indication as to whether the associations with delirium rate were specific, or merely reflecting group differences in general health or health behaviours. These approaches showed that the group differences in delirium for CCBs versus RAS agents, and CCBs versus beta-blockers, were consistent across all three types of cohort. Conversely, whilst there was also a difference in delirium rates between CCB and diuretic cohorts, this finding was not specific to delirium, was not seen in the refined cohort comparison, and was likely attributable to other factors than the AHT classes which defined the cohorts.

Of note, in the main analyses, despite careful baseline matching of cohorts for the recognised factors which influence choice of AHT class, and for delirium risk factors, there continued to be significant differences in the latter during the exposure period. For example, rates of alcohol use disorder, sepsis and use of digitalis glycosides, paralleled group differences in delirium rates (Table 2), even though groups were matched for all these factors at baseline. These findings were unexpected, and their explanation unclear, but they clearly complicate interpretation of the role which AHT class played in the differing rates of delirium observed. This issue was overcome using the refined cohorts (Table 3), but highlights a potential problem for datasets where comparable relevant information during an exposure period is not available.

We found that delirium is commoner with CCBs than RAS agents. This was a robust and relatively large effect. It complements several other observations that RAS agents are associated with lower rates of other neuropsychiatric outcomes including dementia (Kuan et al., 2016; Rouch et al., 2015; van Middelaar et al., 2017; Zhuang et al., 2016), depression (Boal et al., 2016) and bipolar disorder (Shaw et al., 2020). The apparent benefits of RAS agents have been attributed to their effects on the brain angiotensin system, via the cerebral vasculature and central regulation of blood pressure (Fouda et al., 2019; Rocha et al., 2018) and indirectly through impacts on several neurotransmitter systems (Jackson et al., 2018). The lower rate of delirium observed with CCBs compared to beta-blockers extends prior findings that beta-blockers are associated with increased risks of some neuropsychiatric outcomes (Marpillat et al., 2013; Mittal et al., 2017; Mukete et al., 2015), although the data are inconsistent (Ding et al., 2020; Nielsen et al., 2018).

Overall, the present results for delirium are in keeping with a hierarchy of AHTs, in which RAS agents are most beneficial for ‘brain health’, followed by CCBs, with beta-blockers having the most adverse profile. A large observational study of delirium in medical and surgical wards identified a similar ranking (Aloisi et al., 2019). The position of diuretics in the present study and in the other neuropsychiatric literature, is less clear. It should also be noted that a meta-analysis of individual participant data from prospective cohort studies did not identify clear differences between AHT classes in terms of dementia risk (Ding et al., 2020), indicating the need for caution when proposing any such hierarchy.

As well as its strengths, the present study has several limitations. Firstly, even though we controlled for confounding to a much greater degree than in most comparable studies, it is impossible to entirely rule out residual confounding, even for the refined cohorts. One issue is that information on subjects is incomplete, especially for the period before 2007 (see Methods); hence the propensity score matching may also be imperfect. Second, we used the presence of prescriptions separated by at least 2 years as a proxy to identify people who were on the AHT class throughout a two-year period and hence eligible for inclusion in a cohort. This does not exclude the possibility that a patient stopped and restarted medication during this period. Nor do we have any information about adherence to medication nor AHT drug dosage. Third, since patients had to have two prescriptions separated by at least 2 years to enter a cohort, it means that we did not measure cases of delirium which led to the patient having their AHT discontinued. This suggests either that the delirium was mild, or that the AHT was not suspected as a contributory factor. Fourth, except for the deliriogenic drugs excluded in the refined cohorts, we did not control for concurrent medications (e.g. additional AHTs) being taken during the 2 years; hence it is possible that co-prescribing and drug-drug interactions could have contributed to, or counteracted, the observed effects. Fifth, we did not assess whether AHTs *per se* affect risk of delirium, merely whether CCBs are associated with a differential risk compared to other AHT classes. Finally, we cannot necessarily generalise our results to other populations (e.g. younger than 50 years old), or to people with pre-existing cognitive impairment. If sample sizes and data availability permit, further studies will benefit from addressing all these factors, as well as evaluating longer exposure periods, other neuropsychiatric outcomes, and subtypes within each AHT class. For example there is some indication that neuropsychiatric effects may differ between ACEIs and ARBs (Kuan et al., 2016; Marpillat et al., 2013) or amongst CCBs (Hussain et al., 2018).

## Conclusions

We found higher rates of delirium in users of CCBs compared to RAS agents, but lower rates with CCBs compared to beta-blockers. The results are relatively robust, given the large sample sizes, and the use of exclusion criteria, propensity score matching and negative control outcomes. The results further underline the evidence that, compared to other AHTs, RAS agents may be beneficial for various neuropsychiatric phenotypes, and also that beta-blockers may be deleterious. The findings show the value of large pharmacoepidemiological datasets linked to electronic medical records. Equally, the richness of data available on the network allowed us to reveal, and then to minimise, the extent of confounding which can compromise studies of this kind.

## Supplemental Material

Supplementary_Tables_1-3 – Supplemental material for Rates of delirium associated with calcium channel blockers compared to diuretics, renin-angiotensin system agents and beta-blockers: An electronic health records network study Supplemental material, Supplementary_Tables_1-3 for Rates of delirium associated with calcium channel blockers compared to diuretics, renin-angiotensin system agents and beta-blockers: An electronic health records network study by Paul J Harrison, Sierra Luciano and Lucy Colbourne in Journal of Psychopharmacology
