# Supplementary material for: Rates of delirium associated with calcium channel blockers compared to diuretics, renin-angiotensin system agents and beta-blockers: An electronic health records network study
Source: J Psychopharmacol. 2020 Jul 8;34(8):848–55. doi: 10.1177/0269881120936501 (PMC7376629; doi:10.1177/0269881120936501)
Supplement: Supplementary_Tables_1-3 – Supplemental material for Rates of delirium associated with calcium channel blockers compared to diuretics, renin-angiotensin system agents and beta-blockers: An electronic health records network study [file Supplementary_Tables_1-3.pdf]

Supplementary Table 1: Negative control outcomes for unmatched cohorts

	CCBs vs diuretics		CCBs vs RAS agents		CCBs vs beta-blockers	
Benign colonic polyp	3.55% vs 4.16%	**0.85 (0.82-0.87)**	3.70% vs 4.16%	**0.88 (0.86-0.91)**	4.18% vs 3.49%	**1.21 (1.17-1.24)**
Ganglion	0.44% vs 0.57%	**0.77 (0.71-0.84)**	0.50% vs 0.54%	0.92 (0.85-1.00)	0.59% vs 0.41%	**1.44 (1.34-1.56)**
Hallux valgus	0.78% vs 0.93%	**0.84 (0.79-0.89)**	0.87% vs 0.83%	1.05 (0.98-1.12)	0.98% vs 0.69%	**1.41 (1.33-1.49)**
Hernia	4.74% vs 5.45%	**0.86 (0.84-0.89)**	5.74% vs 4.69%	**1.24 (1.20-1.27)**	4.83% vs 5.15%	**0.93 (0.91-0.96)**
Ingrowing nail	0.57% vs 0.76%	**0.75 (0.69-0.80)**	0.62% vs 0.72%	**0.87 (0.80-0.93)**	0.66% vs 0.66%	1.01 (0.95-1.08)
Sebaceous cyst	1.28% vs 1.42%	**0.90 (0.86-0.95)**	1.37% vs 1.37%	1.00 (0.95-1.05)	1.42% vs 1.17%	**1.21 (1.15-1.27)**
Senile keratosis	3.65% vs 3.67%	0.99 (0.96-1.02)	3.73% vs 3.78%	0.98 (0.95-1.02)	3.76% vs 3.47%	**1.08 (1.05-1.12)**
Trigger finger	0.86% vs 0.97%	**0.88 (0.83-0.94)**	0.85% vs 0.97%	**0.88 (0.82-0.94)**	1.02% vs 0.77%	**1.33 (1.25-1.40)**
Otalgia	0.82% vs 1.08%	**0.76 (0.71-0.80)**	1.10% vs 1.08%	1.01 (0.96-1.07)	1.01% vs 0.88%	**1.14 (1.08-1.21)**
Oncholysis	0.37% vs 0.50%	**0.74 (0.68-0.81)**	0.42% vs 0.47%	**0.89 (0.81-0.97)**	0.45% vs 0.45%	0.99 (0.92-1.08)
Viral warts	0.6% vs 0.99%	**0.86 (0.81-0.92)**	0.96% vs 0.98%	0.99 (0.93-1.05)	0.95% vs 0.83%	**1.15 (1.09-1.21)**
Cutaneous abscess	1.05% vs 1.20%	**0.87 (0.83-0.92)**	1.22% vs 1.09%	**1.12 (1.06-1.18)**	1.1% vs 1.00%	**1.11 (1.06-1.17)**
Average		***0.84 (0.79-0.89)***		*0.99 (0.91-1.06)*		***1.17 (1.06-1.27)***

Supplementary Table 2: Negative control outcomes for matched cohorts

	**CCBs vs diuretics**		**CCBs vs RAS agents**		**CCBs vs beta-blockers**	
Benign colonic polyp	3.56% vs 4.28%	**0.82 (0.79-0.85)**	3.71% vs 3.97%	**0.93 (0.89-0.97)**	4.07% vs 3.71%	**1.10 (1.06-1.14)**
Ganglion	0.44% vs 0.52%	**0.84 (0.76-0.93)**	0.50% vs 0.57%	**0.87 (0.79-0.97)**	0.57% vs 0.46%	**1.24 (1.13-1.36)**
Hallux valgus	0.78% vs 0.92%	**0.84 (0.78-0.91)**	0.86% vs 0.95%	**0.90 (0.83-0.98)**	0.90% vs 0.82%	**1.10 (1.02-1.18)**
Hernia	4.73% vs 5.48%	**0.86 (0.82-0.88)**	5.72% vs 4.94%	**1.17 (1.13-1.21)**	4.88% vs 4.86%	1.00 (0.97-1.04)
Ingrowing nail	0.57% vs 0.70%	**0.81 (0.74-0.88)**	0.62% vs 0.68%	0.90 (0.82-1.00)	0.65% vs 0.74%	**0.88 (0.81-0.95)**
Sebaceous cyst	1.28% vs 1.38%	**0.93 (0.88-0.99)**	1.37% vs 1.34%	1.02 (0.95-1.09)	1.42% vs 1.28%	**1.11 (1.05-1.18)**
Senile keratosis	3.66% vs 3.79%	0.96 (0.93-1.0)	3.75% vs 4.04%	**0.92 (0.89-0.96)**	4.04% vs 3.48%	**1.17 (1.13-1.21)**
Trigger finger	0.86% vs 0.89%	0.96 (0.89-1.03)	0.85% vs 0.96%	**0.89 (0.82-0.96)**	0.99% vs 0.85%	**1.17 (1.09-1.25)**
Otalgia	0.82% vs 0.97%	**0.84 (0.78-0.91)**	1.10% vs 1.05%	1.05 (0.98-1.13)	1.00% vs 0.96%	1.03 (0.96-1.11)
Oncholysis	0.37% vs 0.48%	**0.76 (0.69-0.85)**	0.41% vs 0.46%	0.90 (0.80-1.01)	0.43% vs 0.47%	0.91 (0.82-1.01)
Viral warts	0.86% vs 0.96%	**0.90 (0.83-0.96)**	0.97% vs 0.87%	**1.11 (1.02-1.20)**	0.97% vs 0.87%	**1.12 (1.04-1.20)**
Cutaneous abscess	1.04% vs 1.12%	**0.93 (0.86-0.99)**	1.20% vs 1.00%	**1.20 (1.12-1.30)**	1.04% vs 1.04%	1.00 (0.93-1.07)
Average		***0.87 (0.83-0.92)***		*0.99 (0.91-1.06)*		*1.07 (1.00-1.14)*

Supplementary Table 3: Negative control outcomes for refined cohorts

	CCBs vs diuretics		CCBs vs RAS agents		CCBs vs beta-blockers	
Negative control outcomes						
Benign colonic polyp	2.39% vs 2.84%	**0.84 (0.79-0.89)**	2.30% vs 2.59%	**0.89 (0.82-0.96)**	2.72% vs 2.33%	**1.17 (1.10-1.25)**
Ganglion	0.35% vs 0.42%	**0.84 (0.71-0.99)**	0.36% vs 0.45%	**0.81 (0.67-0.98)**	0.41% vs 0.35%	1.17 (1.00-1.37)
Hallux valgus	0.56% vs 0.62%	0.91 (0.80-1.04)	0.60% vs 0.59%	1.02 (0.87-1.19)	0.64% vs 0.60%	1.06 (0.93-1.20)
Hernia	2.78% vs 2.84%	0.98 (0.92-1.04)	2.96% vs 2.61%	**1.14 (1.06-1.22)**	2.84% vs 2.58%	**1.10 (1.04-1.17)**
Ingrowing nail	0.43% vs 0.49%	0.87 (0.75-1.01)	0.43% vs 0.45%	0.96 (0.80-1.15)	0.43% vs 0.50%	0.88 (0.76-1.01)
Sebaceous cyst	1.04% vs 1.13%	0.92 (0.83-1.01)	1.10% vs 1.08%	1.02 (0.91-1.14)	1.10% vs 1.00%	1.10 (1.00-1.21)
Senile keratosis	3.01% vs 3.14%	0.96 (0.90-1.02)	3.00% vs 3.11%	0.96 (0.90-1.03)	3.22% vs 2.91%	**1.11 (1.05-1.18)**
Trigger finger	0.55% vs 0.61%	0.90 (0.79-1.03)	0.52% vs 0.57%	0.90 (0.76-1.05)	0.60% vs 0.53%	1.14 (1.00-1.30)
Otalgia	0.63% vs 0.71%	0.89 (0.79-1.01)	0.78% vs 0.75%	1.04 (0.91-1.19)	0.72% vs 0.68%	1.05 (0.93-1.18)
Oncholysis	0.27% vs 0.33%	0.83 (0.69-1.00)	0.28% vs 0.29%	0.98 (0.78-1.23)	0.28% vs 0.32%	0.86 (0.72-1.03)
Viral warts	0.72% vs 0.76%	0.94 (0.84-1.06)	0.77% vs 0.64%	**1.20 (1.04-1.39)**	0.77% vs 0.73%	1.05 (0.94-1.18)
Cutaneous abscess	0.77% vs 0.79%	0.98 (0.87-1.10)	0.72% vs 0.66%	1.09 (0.94-1.26)	0.72% vs 0.69%	1.05 (0.94-1.18)
Average		***0.90 (0.87-0.94)***		*1.00 (0.93-1.07)*		*1.06 (1.00-1.12)*
